# Can Communication Technologies Reduce Loneliness and Social Isolation in Older People? A Scoping Review of Reviews

**DOI:** 10.3390/ijerph191811310

**Published:** 2022-09-08

**Authors:** Nicola Döring, Melisa Conde, Karlheinz Brandenburg, Wolfgang Broll, Horst-Michael Gross, Stephan Werner, Alexander Raake

**Affiliations:** 1 Media Psychology and Media Design Group, Technische Universität Ilmenau, 98693 Ilmenau, Germany; 2 Electronic Media Technology Group, Technische Universität Ilmenau, 98693 Ilmenau, Germany; 3 Virtual Worlds and Digital Games Group, Technische Universität Ilmenau, 98693 Ilmenau, Germany; 4 Neuroinformatics and Cognitive Robotics Lab, Technische Universität Ilmenau, 98693 Ilmenau, Germany; 5 Audiovisual Technology Group, Technische Universität Ilmenau, 98693 Ilmenau, Germany

**Keywords:** older adults, senior citizens, social inclusion, social wellbeing, interventions, research reviews, technology-mediated communication

## Abstract

Background: Loneliness and social isolation in older age are considered major public health concerns and research on technology-based solutions is growing rapidly. This scoping review of reviews aims to summarize the communication technologies (CTs) (review question RQ1), theoretical frameworks (RQ2), study designs (RQ3), and positive effects of technology use (RQ4) present in the research field. Methods: A comprehensive multi-disciplinary, multi-database literature search was conducted. Identified reviews were analyzed according to the PRISMA (Preferred Reporting Items for Systematic Reviews and Meta-Analyses) framework. A total of *N* = 28 research reviews that cover 248 primary studies spanning 50 years were included. Results: The majority of the included reviews addressed general internet and computer use (82% each) (RQ1). Of the 28 reviews, only one (4%) worked with a theoretical framework (RQ2) and 26 (93%) covered primary studies with quantitative-experimental designs (RQ3). The positive effects of technology use were shown in 55% of the outcome measures for loneliness and 44% of the outcome measures for social isolation (RQ4). Conclusion: While research reviews show that CTs can reduce loneliness and social isolation in older people, causal evidence is limited and insights on innovative technologies such as augmented reality systems are scarce.

## 1. Introduction

The human population is ageing at a fast pace on a global scale. It is estimated that by 2030, one in six people in the world will be aged 60 years or over, and the share of the population aged 60 years and over will increase from 1 billion in 2020 to 1.4 billion in 2030 and 2.1 billion in 2050 [1].

One of the many different challenges of older age is loneliness. Loneliness is defined as an unpleasant subjective experience that emerges when the quantity and/or quality of a person’s actual social relationships does not match their expectations and needs [2]. Being lonely means to feel alone, neglected, abandoned, unwanted, forgotten, and alienated from the social environment. Older people are at risk of feeling lonely for several reasons: changing social roles in older age (e.g., empty nest situation with grown children, retirement from work life), changes in living arrangements (living in residential care), reduced social networks (death of friends and relatives or widowhood), and reduced mobility and less activities outside the home due to more prevalent health issues [3,4,5]. However, it is important to resist the stereotype that all old people must automatically feel lonely, as more and more senior citizens lead meaningful and socially and emotionally fulfilled lives. 

Related to but not identical to loneliness is social isolation. While loneliness is a subjective feeling, social isolation is an objective characteristic of the living situation. Social isolation is defined as an individual’s objective low number or lack of social contacts and relationships with family members, friends, acquaintances, and neighbors [6,7,8]. Social isolation can lead to loneliness. However, not everyone who has a small number of social ties actually feels lonely, and some people with large social networks still suffer from loneliness [9]. 

Feeling lonely and/or being socially isolated are strongly associated with several adverse physical and mental health outcomes: increased blood pressure, heart disease, obesity, diminished immune system functions, increased mortality, depression, anxiety, poorer cognitive functioning, and increased risk of Alzheimer’s disease [10,11,12]. Hence, there is growing concern about battling the “epidemy” of loneliness and social isolation particularly among older adults. 

A variety of targeted interventions aimed at reducing social isolation and loneliness in older people have been developed. With variable degrees of success, these interventions have focused on improving social skills (e.g., through training programs), enhancing social support (e.g., via mentoring programs), increasing opportunities for social interaction (e.g., by organizing social events), and addressing maladaptive social cognitions (e.g., with the help of cognitive behavioral therapy) [13]. 

At the same time, older people themselves try to overcome social isolation and loneliness and develop their own self-directed coping behaviors. Said behaviors can be performed alone or with others. Coping behaviors range from prevention/action (e.g., maintaining hobbies, nurturing social connections, planning and initiating social activities) to acceptance/endurance (e.g., evaluating loneliness and social isolation as inevitable, reframing loneliness and isolation as an opportunity for personal or spiritual growth) [14].

Targeted interventions and self-directed behaviors that are aimed at fighting loneliness and social isolation in older age, are more and more often technology-based. In the digital age, targeted interventions that aim at greater social inclusion of seniors are increasingly designed and delivered as so-called e-interventions using internet technologies, computers, smartphones, custom-made devices and software for seniors, or social robots [3]. At the same time, self-directed behaviors of seniors that aim at greater social inclusion also increasingly appropriate digital communication technologies (CTs) such as texting or videoconferencing among family and friends [12,15].

## 2. State of Research and Review Questions

In recent decades, dozens of primary studies have been conducted that explore and evaluate if and how modern communication technologies can contribute to the social inclusion of older people [3]. CTs can provide opportunities for more frequent and longer interactions with established contacts as well as for finding new communication partners that extend the social network—both processes would reduce objective social isolation. Furthermore, certain communication technologies and usage settings can provide opportunities for particularly deep, meaningful, and emotional communication that provides the experience of a strong interpersonal connection which would, in turn, reduce the feeling of loneliness. The research landscape linking CTs and loneliness and social isolation is so broad and rich that multiple research reviews are available that summarize the many and diverse primary studies on the matter (e.g., [3,16,17]).

Against this background of a growing research landscape, the current paper provides the very first scoping review of reviews aiming to offer a structured overview of the previous body of research in terms of addressed communication technologies, adopted theories, and applied study designs, as well as demonstrated positive effects in terms of the reduction of loneliness and isolation in older people. Our scoping review of reviews covers all previous research reviews without any limit regarding the time period. It includes both reviews covering targeted interventions and self-directed coping behaviors. We have consciously chosen the method of a scoping review of reviews because an earlier outcome-focused systematic review of systematic reviews [3] has shown that primary studies on the matter are very heterogenous and seldomly were randomized controlled trials (RCTs) conducted. Hence, the methodological framework of a scoping review constitutes a better fit to the research aim of providing a complete and comprehensive overview of the research field. 

As opposed to the outcome-focused systematic review, the scoping review [18] covers not only issues of effectiveness of the investigated targeted interventions and self-directed behaviors, but also evaluates the research landscape more broadly by exploring the theories and study designs applied and the communication technologies addressed. By providing a research synthesis based on a scoping review of reviews, we hope to inspire fellow researchers to design and conduct informative studies in the future and help all interested readers to better navigate this growing field of research. 

Our scoping review of reviews answers the following four review questions (RQ):

RQ1: Which types of CTs have been investigated in the context of loneliness and/or social isolation reduction among older people by previous research reviews—considering both targeted interventions and self-directed behaviors?
RQ2: Which theoretical frameworks have been adopted to link CT use with loneliness and/or social isolation reduction among older people by previous research reviews—considering both targeted interventions and self-directed behaviors?
RQ3: Which study designs have been applied to investigate CTs in the context of loneliness and/or social isolation reduction among older people by previous research reviews—considering both targeted interventions and self-directed behaviors?
RQ4: Which effects on loneliness and/or social isolation reduction among older people have been found in connection with the use of CTs by previous research reviews—considering both targeted interventions and self-directed behaviors?

## 3. Results

In the interest of scientific transparency and to reduce the risk of bias, the review methods for this study were established prior to conducting the scoping review of reviews. The procedure follows the Preferred Reporting Items for Systematic reviews and Meta-Analyses extension for Scoping Reviews (PRISMA-ScR) [19] and is pre-registered through the Center of Open Science. The pre-registration and all materials and data are made available via https://osf.io/huc6p/ (accessed on 5 September 2022). The presentation style follows an earlier, thematically unrelated review of reviews in the journal *Healthcare* [20].

### 3.1. Eligibility and Exclusion Criteria

#### 3.1.1. Eligibility Criteria

Following Fujioka et al. (2020) [21], the inclusion of study types was limited to methods-driven reviews (systematic reviews, meta-analyses, scoping reviews, etc.). Eligible reviews had to include at least one primary study analyzing one or more CT-based interventions or self-directed behaviors to reduce loneliness and/or social isolation among older people. Additional review selection criteria were as follows: (a) reviews from any discipline providing data to answer one or more review questions; (b) reviews covering primary studies with participants 55 years of age or older without cognitive impairment; (c) reviews covering primary studies on CT use in the context of targeted interventions and/or self-directed behaviors; (d) reviews covering primary studies on CTs mediating human-to-human communication and/or supporting human–technology communication; and (e) reviews reporting effects on loneliness and/or social isolation reduction.

#### 3.1.2. Exclusion Criteria

Studies that did not apply a systematic review methodology and reviews published in languages other than English were excluded. 

### 3.2. Information Sources and Search Strategy

#### 3.2.1. Electronic Search

The following five electronic databases were searched to identify published review studies: (1) MEDLINE, (2) Institute of Electrical and Electronics Engineers (IEEE) Xplore, (3) Association for Computing Machinery (ACM) Digital Library, (4) Scopus, and (5) PsycINFO. This choice was based on the databases’ reach and diversity of covered scientific fields (medicine, technology, computing, psychology) and was designed to yield the most comprehensive results possible by examining the extent, range, and nature of research activity in line with the aim of scoping reviews [18]. The search was conducted by two researchers between 10 and 11 November 2021.

The search strategy was developed based on preliminary searches and was modified accordingly for each database (e.g., modification of wildcards). The used keywords corresponded to the following four core concepts: (1) loneliness and social isolation, (2) older people, (3) communication technologies, and (4) the publication type research review. A complete list of keywords and examples of search queries can be accessed at https://osf.io/huc6p/ (accessed on 5 September 2022).

#### 3.2.2. Manual Search

A manual search for additional reviews was conducted online and the reference lists of identified studies were screened for additional reviews. 

### 3.3. Study Selection

**Figure 1:**
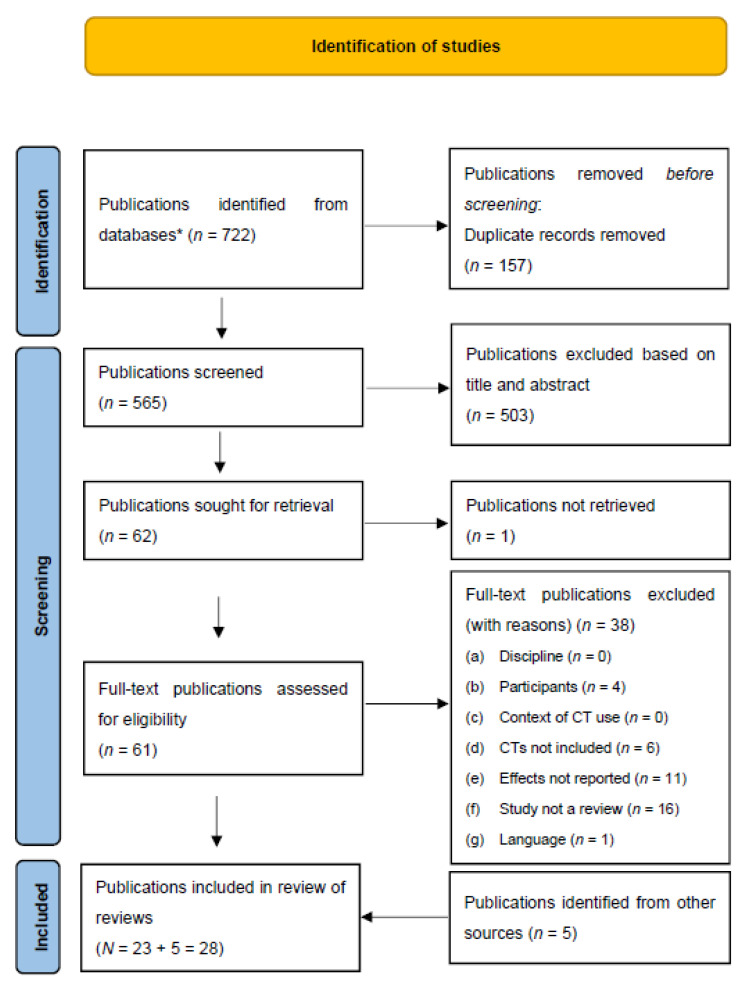
Literature Identification, Screening, and Inclusion for the Scoping Review of Reviews Addressing the Impact of Communication Technologies (CTs) on Loneliness and Social Isolation Among Older Adults. *Note.* Scoping review procedure for literature search, screening, and selection. Figure created following PRISMA-ScR guidelines. Searched databases: (1) MEDLINE, (2) Institute of Electrical and Electronics Engineers (IEEE) Xplore, (3) Association for Computing Machinery (ACM) Digital Library, (4) Scopus, and (5) PsycINFO. Results for each database: MEDLINE (*n* = 113), IEEE (*n* = 14), ACM (*n* = 15), Scopus (*n* = 555), and PsycINFO (*n* = 25).

After the search was conducted across the selected databases, bibliographic information for all search results was exported into the citation management software Citavi 6.8 (Swiss Academic Software GmbH). Duplicates were removed and the number of results was recorded. Titles and abstracts were screened against the inclusion and exclusion criteria and, subsequently, full texts of the selected reviews were retrieved and assessed. The screening process was conducted by the second author and checked by the first author, and was reported following PRISMA-ScR guidelines [19] in Figure 1.

### 3.4. Data Collection and Charting

All *N* = 28 included publications were reviewed and charted by one researcher (second author) in a predesigned document. The completed charting form was examined by an expert from the field (first author). Only minor inconsistencies appeared that were resolved by discussion. The double-checked *final charting document* (accessible via https://osf.io/huc6p/ (accessed on 5 September 2022) contains the following variables for each of the included research reviews: (a) citation (authors, year of publication, title, journal name, page number, and DOI); (b) countries of authors; (c) academic disciplines of authors; (d) number of citations (Google Scholar); (e) type of review; (f) quality assessment; (g) time period covered (according to range of publication years of primary studies); (h) number of included primary studies; (i) total sample; (j) number of primary studies addressing CTs; (k) context of CT use (targeted intervention or self-directed behavior); (l) type of studied CT as classified in the included reviews (type of communication *device*: computer [general use], [landline] telephone, smartphone [general use], social robot, tablet [general use]; type of communication *system/application*: augmented reality/virtual reality (AR/VR) system, chat/messaging application, email, internet [general use], social networking site, videoconference system, other communication system); (m) theoretical framework of review (present or not present); (n) study design (qualitative, quantitative or mixed-methods); (o) outcome measures for loneliness and/or social isolation. The variables are used to describe the previous research reviews and to answer the four review questions.

In addition, the methodological quality of all included research reviews was assessed using the instrument AMSTAR 2 (A Measurement Tool to Assess Systematic Reviews, version 2) [22]. Given that not all reviews come from the field of health research and therefore do not follow the guidelines for medical reporting (which is the focus of AMSTAR 2), we adopted the 7 items (out of 16) determined by Shea et al. (2017) to be critically important for the validity of any research review. Our complete *quality assessment document* (accessible via https://osf.io/huc6p/ (accessed on 5 September 2022) includes the following critical items: (a) item 2: establishment of methods previous to study conduction; (b) item 4: literature search strategy; (c) item 7: excluded studies; (d) item 9: risk-of-bias assessment; (e) item 11: statistical methods for meta-analysis; (f) item 13: risk of bias in individual studies; (g) item 15: publication bias assessment. As suggested by Shea et al. (2017), we did not compute an overall score by combining all individual item ratings. Instead, we first assessed which items were relevant for each specific review (e.g., meta-analysis items were only applied to reviews including meta-analyses; risk-of-bias items were only applied to reviews including RCTs) and rated it accordingly. We then classified the reviews regarding overall confidence in the results. Our classification was obtained by assigning values to the fulfilled relevant quality criteria (presented as percentages). The quality of the included research reviews was classified as follows: (1) high = 75%–100% of all critical 7 items fulfilled, (2) moderate = 50%–74%, (3) low = 25%–49%, and (4) critically low = 0%–24%. Out of all *N* = 28 included reviews, 3 (11%) were considered to have high quality, 10 (36%) to have moderate quality, 3 (11%) to have low quality, and 12 (43%) to have critically low quality (see Table 1). While less than half of the reviews in this study had critically low quality, in other research fields, the majority of research reviews (74%–99%) showed critically low quality [23,24,25,26,27]. Regardless of quality, all reviews were included in this scoping review of reviews to cover the complete state of research.

### 3.5. Methods of Data Analysis

#### 3.5.1. Reporting of Descriptive Characteristics 

The general characteristics of the *N* = 28 included reviews are displayed in Table 1. Focus was placed on the primary studies addressing the use of communication technologies to reduce loneliness and/or social isolation. A total of 248 primary studies addressing CTs (after removing duplicates) with over 71,000 participants were considered for the present study. The precise total number of included participants is unclear as some reviews cover qualitative primary studies that do not report their exact sample sizes. Included reviews report on primary studies published between 1970 and 2020, spanning 50 years of research. Each included review covers between 1–52 primary studies addressing CTs and a total sample in the range of 36–8895 participants (see Table 1). 

#### 3.5.2. Statistical Analysis

Quantitative variables were coded as 1 (present) and 0 (not present) while open coding was used for qualitative variables. Variables for each review question were coded as follows: RQ1 (types of CT): Each technology was coded quantitatively. RQ2 (theoretical frameworks): Theories were coded openly. RQ3 (study designs): Designs were grouped and coded quantitatively. Additionally, the number of primary studies in each design group was counted. RQ4 (effects on loneliness and/or social isolation): The number of outcome measures for loneliness and/or social isolation within each review was coded quantitatively while the reported effects were described qualitatively. 

This review provides the sum of outcome measures for loneliness and the sum of outcome measures for social isolation instead of giving all reviews the same weight. Therefore, if a review comprises 10 individual primary studies and reports 5 outcome measures for loneliness and 5 outcome measures for social isolation, we decided to report said outcome measures separately instead of only reporting the review result as one individual result. This decision was made given that the number of included studies analyzing CT impact differed greatly from one review to another (between 1 and 41 studies per review). Therefore, reporting individual outcome measures as opposed to reporting 28 general review results with apparently the same weight was considered a more transparent and comprehensive approach.

The reported outcomes come from one of two sources, depending on the type of studies covered by the included reviews:

- If an included review covered only primary studies addressing CT-related interventions and/or self-directed behaviors, the general conclusions of the review were used to report the effects.
- If an included review covered both CT-related and non-CT-related interventions and/or self-directed behaviors, only the technology-related effects were used to report the effect. The aforementioned technology-related effects were extracted from the results section, individual tables, or specific sections in the overall conclusions of the respective included reviews.

We classified the outcomes as “positive effect” if a decrease in loneliness and/or social isolation or an association with lower levels of loneliness and/or social isolation was reported and as “negative effect” if an increase in loneliness and/or social isolation or an association with higher levels of loneliness and/or social isolation was reported. We coded “no effect” if no significant variation in loneliness and social isolation was reported and as “unclear effect” if no general conclusion related to effects of technology use on loneliness and/or social isolation was reported or the review stressed conflicting results (mixture of positive and no effects). 

All quantitative data are presented using absolute frequencies and/or percentages and all qualitative data are presented in narrative form. Given the nature of the scoping review, only descriptive statistics are used to present the data.

## 4. Results

### 4.1. Types of Communication Technologies

To answer RQ1, a count of the technologies and the number of reviews covering them was performed and revealed the following main results: Internet and computer were the most researched communication technologies (23 reviews or 82% each), followed by videoconference systems such as Zoom or Skype (16 reviews, 57%), email (13 reviews, 46%), telephone (12 reviews, 43%), and social robot (10 reviews, 36%). Augmented reality (AR) or virtual reality (VR) systems were covered by only one of the 28 included reviews (4%; see Table 2). 

Technologies classified as “other” (14 reviews) usually referred to custom-made systems especially designed with older people’s needs in mind. The following systems were covered by the included reviews: *PRISM* (Personal Reminder Information and Social Management) is a special software for seniors created to support social connectivity, memory, and leisure activities [34,43,48,55]. *Koffee Klatch* is an asynchronous, peer-led support chat room that provides an opportunity for older women to chat about various health topics in the presence of healthcare experts [48]. *LEAP* (Living, Eating, Activity, and Planning through retirement) is an online social platform that helps the elderly improve their diet, physical activity, and social connections as a way to enhance healthy aging [34]. *Elder Tree*, another online social platform, was designed to support social connectedness, driving, caregiving, medication management, and fall prevention as well as reduce the physical, emotional, and financial burdens of the elderly and their families [34]. *Senior App Suite*, a system created for assisting seniors’ personal independence and social inclusion, integrates mobile computing combined with web and service-oriented technologies [43]. Finally, the *GezelschApp* is a mobile application created to reduce social isolation and loneliness among older adults by giving them access to a homepage with six features: an inbox for messages, news, activities, information, tips, and friends [43]. 

**Figure 2:**
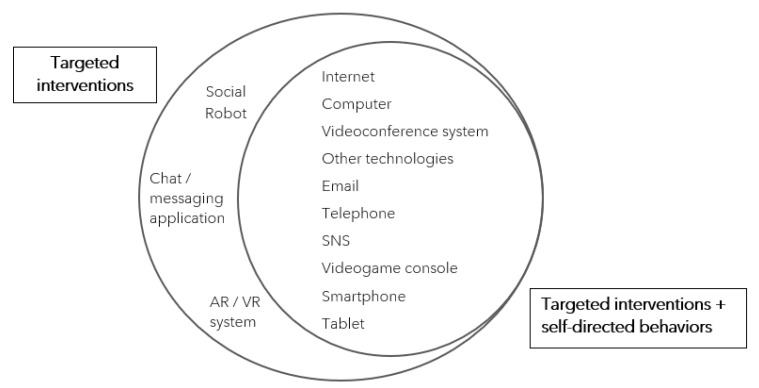
Investigated CTs According to Context of Use as Represented in the Included Research Reviews. *Note.* Abbreviations: (SNS) social networking site, (AR/VR) augmented reality/virtual reality system. Technologies are grouped based on the context in which they were studied: exclusively in targeted interventions or in targeted interventions and self-directed behaviors. *N* = 28 included research reviews.

Most studied technologies were used in the context of targeted interventions. Moreover, only two of the included reviews covered both targeted interventions and self-directed behaviors—[28] and [32]. Among all covered technologies, three were studied exclusively in the context of targeted interventions, namely social robots, chat/messaging applications, and AR/VR systems (see Figure 2).

### 4.2. Theoretical Frameworks

In regard to RQ2, related to which theoretical frameworks have been used to link CT usage with reduced loneliness and/or social isolation of older people, this review determined that out of the *N* = 28 included reviews, only one made use of a general theory at the review level (Table 3).

Masi et al. (2011) [50] hypothesized in their meta-analysis that—given the established centrality of social cognition to the experience of loneliness [56,57]—interventions that address maladaptive social cognition would have a greater impact on loneliness reduction than those that address social skills, social support, or opportunities for social interaction. Their meta-analytic empirical results supported the *Social Cognitive Theory* developed by psychologist Albert Bandura (1986) [58].

Most included reviews did not provide any theoretical framework and they acknowledged this as one of their main limitations. 

### 4.3. Study Designs

To answer RQ3, a count of study designs used in the included reviews was undertaken. Study designs were divided into four groups: qualitative designs, quantitative-observational designs, quantitative-experimental designs, and mixed-methods designs (see Table 4). 

*Qualitative designs* were adopted in primary studies covered by 16 reviews. The included review with the highest number of included qualitative primary studies contained 24 qualitative primary studies [28] and the included review with the smallest number of qualitative primary studies contained one [36]. The most widely used qualitative methods of data collection were in-depth and/or semi-structured interviews, focus group discussions, and participant observations. Data analysis was usually conducted using a descriptive thematic method in which the collected data were organized and presented according to prominent themes. Outcome measures of qualitative primary studies entailed, for example, qualitative assessments of the perceived benefits of general internet use to overcome loneliness and/or social isolation, perceptions of social presence when using CTs, reports of reconnecting with family through off-the-shelf applications, and factors that impact the easy adoption of digital devices and software among older adults [28].

*Quantitative-observational designs* were used in primary studies covered by 19 reviews. Gardiner et al. (2018) [39] summarized the highest number of quantitative-observational primary studies (21), while Shah et al. (2021) [55] covered the smallest number (1). Surveys (cross-sectional and longitudinal) were the most used data collection methods and data analysis was performed using descriptive and inferential statistics to determine the correlations between CT use and loneliness and/or social isolation. Outcome measures of quantitative-observational primary studies included associating technology use with levels of loneliness and/or social isolation or comparing loneliness and/or social isolation levels between groups of users and non-users of technology.

*Quantitative-experimental designs* were applied in primary studies covered by almost all included reviews (26/28). Masi et al. (2011) [50] covered the highest number of quantitative primary studies (50) and Brimelow and Wollin (2017) [29] the lowest number (2). RCTs and quasi-experimental designs were the most commonly included types of quantitative-experimental studies. Outcome measures for quantitative-experimental designs were changes in loneliness and/or social isolation levels after the interventions. 

*Mixed-methods designs* were applied in the primary studies of 10 reviews. Of those reviews, Gasteiger et al. (2021) [40] and Heins et al. (2021) [43] covered the highest number of mixed-methods studies (10 each), while Poscia et al. (2018) [54] covered the lowest number (1). The most used methods of data collection were combinations of surveys and qualitative interviews. Data analysis was performed through statistical and thematic analysis, respectively. 

For all quantitative studies, *outcome measures for loneliness and social isolation* were mostly obtained through the application of standardized scales. The most used *psychometric scales for loneliness* were the UCLA (University of California, Los Angeles, CA, USA) Loneliness Scale [7] and the De Jong Gierveld Loneliness Scale [59]. The UCLA Loneliness Scale contains items such as: “I am no longer close to anyone”, “I feel isolated from others”, and “No one really knows me well”. The De Jong Gierveld Loneliness Scale contains items such as: “I often feel rejected”, “I miss having a really close friend”, and “I experience a general sense of emptiness”. Hence, outcome measures for loneliness mainly address feelings of social exclusion and aloneness.

The used *psychometric scales for social isolation* were diverse and none was used in a generalized manner across reviews. Measures included the Friendship Scale [60], the Lubben Social Network Scale (LSNS) [61], and the Duke Social Support Index (DSSI) [62]. Typical for a social isolation measure, the LSNS, for example, focuses not so much on feelings of disconnectedness, but asks about an objective number of contacts: “How many relatives do you see or hear from at least once a month?” and “How many of your friends do you see or hear from at least once a month?”. The DSSI measures social isolation with similar questions such as “How many times during the past week did you spend time with someone who does not live with you, that is, you went to see them or they came to visit you or you went out together?” and “About how often did you go to meetings of clubs, religious meetings, or other groups that you belong to in the past week?”. 

### 4.4. Effects on Loneliness and Social Isolation

To answer RQ4, reported outcome measures for loneliness and social isolation are summarized in a table (see Table 4 above) as well as in two overview tables. As shown in Table 5, from a total of 62 CT-related outcome measures for loneliness reduction reported across the included 28 reviews, 55% of the outcome measures (34/62) showed a positive effect of CT use, 12% (7/62) no effect, 34% (21/62) unclear effects, and 0% negative effects. 

From a total of 41 CT-related outcome measures from individual studies reported for social isolation reduction, 44% (18/41) point to a positive effect of technology use. However, slightly more than half (55%) revealed no effect (5/41) or unclear effects (18/41) (See Table 6).

While the majority of reported outcome measures on loneliness (55%) point to positive effects in terms of loneliness reduction through communication technology use among older people, the minority of reported outcome measures on social isolation (44%) confirm positive effects.

## 5. Discussion

### 5.1. Interpretation of Main Results

Several types of CTs have been studied in an effort to understand their potential positive impact on the reduction of loneliness and social isolation among older adults (RQ1). As evidenced by this scoping review of reviews, internet and computer use are the types of CT covered by most of the included reviews. However, the vagueness of the concepts and related measures makes it impossible to clearly understand which type of internet and computer use was associated with a reduction in loneliness or isolation. The CTs that were more narrowly defined and measured, such as the use of videoconference systems, email, social networking sites, or videogame consoles, were most often covered in previous reviews in the context of targeted interventions and only seldom in the context of self-directed behaviors. As more and more older adults adopt CTs in their everyday lives, it becomes more relevant to analyze CT use in relation to the reduction of loneliness and/or social isolation [63]. However, the most innovative CTs that are not yet widely available on the consumer market, such as advanced social robots or expensive VR/AR systems, can so far only be investigated in the context of targeted interventions when older participants are equipped with the technology by the research team. So far, social robots have been investigated much more often as tools for loneliness or isolation prevention and reduction [64,65] as opposed to VR/AR systems. Furthermore, efforts to study custom-made technologies that aim at enhancing older adults’ social inclusion through functionalities that cater to their age-related characteristics, lifestyles, and socialization needs were very prevalent in the included reviews. Applications such as PRISM, Koffee Klatch, LEAP, Elder Tree, Senior App Suite, and GezelschApp, among others, were covered by 50% of the included reviews. Such “other technologies” were often developed in a human-centered design process involving older adults as key stakeholders [66]. However, their impact is limited because the respective communication technologies are not available outside the research projects. 

The lack of an overarching theoretical framework linking communication technology use and loneliness or social isolation reduction was evident in all reviews except one (RQ2). Although some reviews reported which theories were used to create certain individual targeted interventions, no comprehensive frameworks at the review level were used when summarizing CTs and their impact on loneliness and/or social isolation among older adults which is a limitation acknowledged by the reviews and highlighted in the methodology literature (e.g., [67]).

Most of the included research reviews covered quantitative and even experimental designs, while qualitative and mixed-methods designs were not that prevalent (RQ3). Still, causal evidence is limited as primary studies as well as extant review studies suffer from limited methodological quality, as our AMSTAR 2 analysis of the included reviews has demonstrated. 

Positive effects of CT use were shown in 55% of the outcome measures for loneliness and 44% of the outcome measures for social isolation presented in the reviews summarized in this scoping review of reviews. Furthermore, there are some slight differences between the number of outcome measures showing positive effects compared to no effects or unclear effects from one study design to another; however, said differences are not substantial enough to be included in the global analysis of the study (RQ4). This can be characterized as an ambivalent overall result, as in roughly half of the cases, the intended positive effect of communication technology was not reached. This could be caused by deficits in theoretical assumptions, in the design of the communication technologies, or the usage of the CTs and/or study designs and instruments. The slightly more positive overall result for loneliness could indicate, for example, that issues of loneliness were addressed more sufficiently by technology use and/or that changes in loneliness were measured more precisely by adopting mainly one established instrument, namely the UCLA Loneliness Scale. Divergent and vague definitions of loneliness and social isolation, the interchangeable use of both terms, and unclear empirical differentiation, particularly in qualitative studies, are known problems in the research field [3,17] that could partly explain unclear effects. 

In general, most reviews conclude that further research is needed in order to decisively confirm the causal effects of CT use regarding the reduction of loneliness and/or social isolation among older adults. A call for conducting methodologically and theoretically robust studies is made in most reviews. However, conducting studies with older adults presents a specific set of challenges that need to be tackled. Recruiting older people can be difficult given that they are considered a hard-to-reach group. Additionally, age-related issues (such as declining health, compressed life expectancy, mobility issues, etc.) cause studies involving older adults to have higher attrition levels. Furthermore, conceptual issues regarding concise classifications of CT use and a clear differentiation between loneliness versus social isolation need to be resolved. More theory work linking the characteristics of different communication technologies with processes of loneliness and social isolation reduction taking into consideration different types of communication partners, interaction tasks, and topics is necessary. 

### 5.2. Limitations and Strengths

This scoping review of reviews is not without its limitations. Firstly, only English-language reviews were considered during the screening process, possibly causing the exclusion of relevant reviews published in other languages.

Additionally, there were some minor deviations from the exclusion criteria, namely age limit (some primary studies included a very small number of individuals under 55 years of age) and cognitive state (some primary studies included a very small number of individuals with dementia). However, these deviations were so minor that excluding the whole review would have created an even stronger bias. 

The classification of CTs (according to main communication devices or main communication systems/applications) was adopted from the included reviews and has to be characterized as pragmatic. It is not an analytical classification; hence, the categories come with some vagueness and overlap, particularly in the broad categories of general “internet” use and general “computer” use. This problem could not be resolved in this review of reviews because it goes back to the vague measurement of communication technology use in the primary studies.

Unfortunately, primary studies also are often vague about the key outcome measures of “loneliness” and/or “social isolation” reduction. Both terms were sometimes used interchangeably and therefore some inconsistencies in the reported outcome measures may have occurred, particularly in the qualitative studies. 

Finally, the AMSTAR 2 quality assessment tool is not an optimal fit for scoping reviews given that the objective of a scoping review is to include a broad spectrum of study designs and is not limited to systematic reviews and meta-analyses (which is the main focus of AMSTAR 2). However, the aforementioned tool is the best established and most used instrument for assessing research review quality and was therefore adopted for this scoping review of reviews.

### 5.3. Outlook on Future Research and Practice

Among the research gaps identified in the present scoping review of reviews are theory development, the provision and use of validated measures particularly of social isolation, longitudinal study designs, and RCTs establishing causality. 

Furthermore, there was a notable absence of coverage of self-directed behaviors within the included reviews. More insights in said behaviors are needed to better understand how current and future generations of seniors adopt and use off-the-shelf communication technologies in self-directed ways to overcome loneliness and social isolation. 

Along the same lines, a focus on participatory and human-centered designs could benefit the development of future technologies being created especially for seniors. Although several custom-made CTs were developed and evaluated in the context of targeted interventions, these CTs are not commercially available and cannot be used outside respective research settings. Future projects, hence, should have an eye on the sustainability of innovative technological solutions and interventions. 

Finally, the adverse physical and psychological outcomes associated with loneliness and social isolation in old age make this study relevant for medical professionals and health institutions. With technologies becoming mainstream communication tools, an opportunity arises to understand which CTs could produce holistic positive effects by helping maintain social contacts and supporting societal participation of older adults. Additionally, the integrative potential of certain CTs could be harnessed through their adoption in clinical settings, assisted living facilities, and private homes where residents are unable to participate in social activities due to limited mobility or health issues. 

## 6. Conclusions

This scoping review of reviews—the very first to provide a complete systematic overview of the research field by mapping all studied CTs, theories, and study designs in the context of targeted interventions and self-directed behaviors—confirmed the positive associations of CT use with loneliness and social isolation reduction among older people. There is, however, limited causal evidence and a noticeable absence of innovative technologies in the reviewed literature. Nevertheless, the presented summary of established effects, as well as a clear assessment of the methodological quality of the included reviews, offer a solid starting point for further and future research in the field.

## Figures and Tables

**Table 1:** Description of Included Reviews Addressing the Impact of CTs on Loneliness and Social Isolation Among Older Adults.

Ref.	Authors	Range of Publication Years of Primary Studies	Number of Primary Studies Addressing CTs	Total Sample of Primary Studies Addressing CTs	AMSTAR 2 Quality Score of Review
[28]	Baker et al. (2018)	2000–2016 ^1^	36	N/A	4
[29]	Brimelow and Wollin (2017)	1996–2013	4	233	4
[30]	Casanova et al. (2021)	2002–2019	11	953	2
[31]	Cattan et al. (2005)	1970–2002	7	745	3
[32]	Chen et al. (2021)	2007–2018	52	5844	4
[33]	Chen and Schulz (2016)	2002–2015	30	N/A	2
[34]	Choi and Lee (2021)	2003–2019	21	1323 ^2^	2
[35]	Choi et al. (2012)	2001–2011	6	373	2
[36]	Cohen-Mansfield and Perach (2015)	1996–2011	12	694	2
[37]	Dickens et al. (2011)	1976–2009	6	767	2
[38]	Franck et al. (2016)	2010–2011	1	36	2
[39]	Gardiner et al. (2018)	2003–2016	9	N/A	4
[40]	Gasteiger et al. (2021)	2003–2020	29	632	3
[41]	Gorenko et al. (2021)	2007–2018	19 ^3^	N/A	4
[42]	Hagan et al. (2014)	2000–2012	6	439	4
[43]	Heins et al. (2021)	2005–2020	36	N/A	1
[44]	Ibarra et al. (2020)	until 2020 ^1^	25	N/A	4
[45]	Ibrahim et al. (2021)	1978–2018	4	162	4
[46]	Isabet et al. (2021)	2003–2018	24	377	4
[47]	Khosravi and Ghapanchi (2016)	2002–2013	41 ^4^	N/A	4
[48]	Khosravi et al. (2016)	2002–2015	34	8895	4
[49]	Li et al. (2018)	2009–2017	10	382	2
[50]	Masi et al. (2011)	1982–2009	8	410	3
[51]	Morris et al. (2014)	2000–2013	18	2343	2
[52]	Noone et al. (2020)	2010–2020	3	201	1
[53]	O’Rourke et al. (2018)	1984–2014	4	N/A	4
[54]	Poscia et al. (2018)	2012–2015	4	319	2
[55]	Shah et al. (2021)	2010–2019	6	646	1

*Note*. Abbreviations: (Ref.) number according to reference list, (CTs) communication technologies. ^1^ Years included in the search strategy. ^2^ Total sample of studies with quantitative and mixed methods design. No sample size information available for qualitative studies. ^3^ 7 studies addressing loneliness and/or social isolation. ^4^ 8 studies addressing social isolation. Overall quality score according to AMSTAR 2: (1) high, (2) moderate, (3) low, and (4) critically low. N/A = Data not available. *N* = 28 included research reviews.

**Table 2:** Investigated CTs and Context of Use as Represented in the Included Research Reviews (Descending Order of Prevalence).

CTs	Total No. of Reviews (No. of Reviews with SDB)	Reviews
Internet	23 (2)	[28] *, [29,30,31], [32] *, [33,34,35,36,37,39,41,42,43,44,45,47,48,50,51,53,54,55]
Computer	23 (1)	[28] *, [29,30,31,33,34,35,36,37,39,40,41,43,44,45,47,48,50,51,52,53,54,55]
Videoconference system	16 (2)	[28] *, [29], [32] *, [33,34,36,39,41,42,43,44,48,52,53,54,55]
Other technologies	14 (2)	[28] *, [32] *, [33,34,39,40,42,43,44,45,48,51,54,55]
Email	13 (2)	[28] *, [30], [32] *, [33,34,35,36,43,44,45,50,53,54]
Telephone	12 (1)	[28] *, [31,33,36,37,39,41,43,44,45,50,53]
SNS	11 (2)	[28] *, [30], [32] *, [33,34,39,41,43,44,48,55]
Videogame console	10 (1)	[28] *, [29,33,34,38,42,43,48,49,51]
Social robot	10 (0)	[29,34,39,40,42,46,47,48,53,54]
Smartphone	8 (2)	[28] *, [30], [32] *, [33,34,43,44,52]
Tablet	7 (1)	[28] *, [30,33,34,43,44,52]
Chat/messaging app	6 (0)	[33,34,43,44,51,55]
AR/VR system	1 (0)	[48]

*Note*. Abbreviations: (CTs) communication technologies, (No.) number, (SDB) self-directed behaviors, (SNS) social networking site, (AR/VR) augmented reality/virtual reality system. All reviews include targeted interventions. Reviews marked with (*) include targeted interventions and self-directed behaviors. CTs presented in descending order according to total number of reviews covering the respective technology. *N* = 28 included research reviews.

**Table 3:** Theoretical Frameworks Linking CT Use with Reduced Loneliness and Social Isolation Among Older Adults as Represented in the Included Research Reviews.

Review	Theoretical Framework	No. of Primary Studies Applying Theoretical Framework	Link between CT Use and Reduced Loneliness and/or Isolation
Masi et al. (2011) [50]	Social Cognitive Theory (SCT)	1 (social cognitive training through telephone calls)	Interventions that address maladaptive social cognition will have a greater impact than those addressing social skills, social support, and social interaction. Outcome measures for loneliness were obtained by applying the 10-item UCLA Loneliness Scale. No outcome measures for social isolation were reported.

*Note*. Abbreviations: (No.) number, (CT) communication technology. *N* = 28 included research reviews.

**Table 4:** Study Designs and Reported Effects of the Use of CTs on Loneliness and Social Isolation Among Older Adults as Represented in the Included Research Reviews.

Qualitative Designs (16 Reviews)					
Ref.	Author(s)	CTs	Primary Studies with Qualitative Design	AMSTAR 2 Quality Score of Review	Effects on L/SI
[28]	Baker et al. (2018)	Telephone, smartphone, tablet, computer, videogame console, videoconference system, email, SNS, internet, other technologies	24	4	L = n/a SI = unclear effect
[33]	Chen and Schulz (2016)	Telephone, smartphone, tablet, computer, videogame console, messaging app, videoconference system, email, SNS, internet, other technologies	14	2	L = unclear effect SI = positive effect
[43]	Heins et al. (2021)	Telephone, smartphone, tablet, computer, videogame console, messaging app, videoconference system, email, SNS, internet, other technologies	14	1	L = unclear effect SI = unclear effect
[53]	O’Rourke et al. (2018)	Telephone, computer, social robot, videoconference system, email, internet, other technologies	12	4	L = n/a ^1^ SI = n/a ^1^
[39]	Gardiner et al. (2018)	Telephone, computer, social robot, videoconference system, SNS, internet, other technologies	10	4	L = positive effect SI = n/a
[32]	Chen et al. (2021)	Smartphone, videoconference system, email, SNS, internet, other technologies	8	4	L = positive effect SI = positive effect
[40]	Gasteiger et al. (2021)	Computer, social robot, other technologies	7	3	L = positive effect SI = n/a
[44]	Ibarra et al. (2020)	Telephone, smartphone, tablet, computer, messaging app, videoconference system, email, SNS, internet, other technologies	6	4	L = positive effect SI = no effect
[34]	Choi and Lee (2021)	Smartphone, tablet, computer, social robot, videogame console, messaging app, videoconference system, email, SNS, internet, other technologies	5	2	L = unclear effect SI = positive effect
[49]	Li et al. (2018)	Videogame console	5	2	L = positive effect SI = n/a
[54]	Poscia et al. (2018)	Telephone, smartphone, tablet, computer, social robot, videogame console, AR/VR system, messaging app, videoconference system, email, SNS, internet, other technologies	5	2	L = positive effect SI = n/a
[46]	Isabet et al. (2021)	Social robot	5	4	L = unclear effect SI = unclear effect
[48]	Khosravi et al. (2016)	Computer, social robot, videogame console, AR/VR system, videoconference system, SNS, internet, other technologies	4	4	L = positive effect SI = positive effect
[41]	Gorenko et al. (2021)	Telephone, computer, videoconference system, SNS, internet, other technologies	3	4	L = positive effect ^2^ SI = positive effect ^2^
[45]	Ibrahim et al. (2021)	Telephone, computer, email, internet, other technologies	3	4	L = no effect SI = unclear effect
[36]	Cohen-Mansfield and Perach (2015)	Telephone, computer, videoconference system, email, internet	1	2	L = positive effect SI = n/a
				**Total**	Positive effect: L (9), SI (5) No effect: L (1), SI (1) Unclear effect: L (4), SI (4)
**Quantitative-Observational Designs (19 Reviews)**					
**Ref.**	**Author(s)**	**CTs**	**Primary Studies with Quantitative-Observational Design**	**Quality Score**	**Effects on L/SI**
[39]	Gardiner et al. (2018)	Telephone, computer, social robot, videoconference system, SNS, internet, other technologies	21	4	L = positive effect SI = n/a
[32]	Chen et al. (2021)	Smartphone, videoconference system, email, SNS, internet, other technologies	20	4	L = positive effect SI = positive effect
[53]	O’Rourke et al. (2018)	Telephone, computer, social robot, videoconference system, email, internet, other technologies	16	4	L = n/a ^1^ SI = n/a ^1^
[48]	Khosravi et al. (2016)	Computer, social robot, videogame console, AR/VR system, videoconference system, SNS, internet, other technologies	15	4	L = positive effect SI = positive effect
[44]	Ibarra et al. (2020)	Telephone, smartphone, tablet, computer, messaging app, videoconference system, email, SNS, internet, other technologies	14	4	L = positive effect SI = no effect
[33]	Chen and Schulz (2016)	Telephone, smartphone, tablet, computer, videogame console, messaging app, videoconference system, email, SNS, internet, other technologies	10	2	L = unclear effect SI = positive effect
[28]	Baker et al. (2018)	Telephone, smartphone, tablet, computer, videogame console, videoconference system, email, SNS, internet, other technologies	9	4	L = n/a SI = unclear effect
[46]	Isabet et al. (2021)	Social robot	8	4	L = unclear effect SI = unclear effect
[54]	Poscia et al. (2018)	Telephone, smartphone, tablet, computer, social robot, videogame console, AR/VR system, messaging app, videoconference system, email, SNS, internet, other technologies	7	2	L = positive effect SI = n/a
[51]	Morris et al. (2014)	Computer, videogame console, messaging app, internet, other technologies	6	2	L = unclear effect SI = unclear effect
[34]	Choi and Lee (2021)	Smartphone, tablet, computer, social robot, videogame console, messaging app, videoconference system, email, SNS, internet, other technologies	5	2	L = unclear effect SI = positive effect
[36]	Cohen-Mansfield and Perach (2015)	Telephone, computer, videoconference system, email, internet	5	2	L = positive effect SI = n/a
[40]	Gasteiger et al. (2021)	Computer, social robot, other technologies	5	3	L = positive effect SI = n/a
[49]	Li et al. (2018)	Videogame console	5	2	L = positive effect SI = n/a
[42]	Hagan et al. (2014)	Social robot, videogame console, videoconference system, internet, other technologies	2	4	L = unclear effect SI = n/a
[43]	Heins et al. (2021)	Telephone, smartphone, tablet, computer, videogame console, messaging app, videoconference system, email, SNS, internet, other technologies	2	1	L = unclear effect SI = unclear effect
[45]	Ibrahim et al. (2021)	Telephone, computer, email, internet, other technologies	2	4	L = no effect SI = unclear effect
[47]	Khosravi and Ghapanchi (2016)	Computer, social robot, internet	1	4	L = n/a SI = positive effect
[55]	Shah et al. (2021)	Computer, messaging app, videoconference system, SNS, internet, other technologies	1	1	L = positive effect SI = n/a
				**Total**	Positive effect: L (9), SI (5) No effect: L (1), SI (1) Unclear effect: L (6), SI (5)
**Quantitative-Experimental Designs (26 Reviews)**					
**Ref.**	**Author(s)**	**CTs**	**Primary Studies with Quantitative-–Experimental Design**	**Quality Score**	**Effects on L/SI**
[50]	Masi et al. (2011)	Telephone, computer, email, internet	50	3	L = no effect SI = no effect
[37]	Dickens et al. (2011)	Telephone, computer, internet	32	2	L = no effect SI = n/a
[31]	Cattan et al. (2005)	Telephone, computer, internet	30	3	L = no effect SI = no effect
[36]	Cohen-Mansfield and Perach (2015)	Telephone, computer, videoconference system, email, internet	28	2	L = positive effect SI = n/a
[53]	O’Rourke et al. (2018)	Telephone, computer, social robot, videoconference system, email, internet, other technologies	25	4	L = n/a ^1^ SI = n/a ^1^
[45]	Ibrahim et al. (2021)	Telephone, computer, email, internet, other technologies	18	4	L = no effect SI = unclear effect
[48]	Khosravi et al. (2016)	Computer, social robot, videogame console, AR/VR system, videoconference system, SNS, internet, other technologies	15	4	L = positive effect SI = positive effect
[51]	Morris et al. (2014)	Computer, videogame console, messaging app, internet, other technologies	12	2	L = unclear effect SI = unclear effect
[30]	Casanova et al. (2021)	Smartphone, tablet, computer, email, SNS, internet	11	2	L = positive effect SI = n/a
[43]	Heins et al. (2021)	Telephone, smartphone, tablet, computer, videogame console, messaging app, videoconference system, email, SNS, internet, other technologies	10	1	L = unclear effect SI = unclear effect
[32]	Chen et al. (2021)	Smartphone, videoconference system, email, SNS, internet, other technologies	8	4	L = positive effect SI = positive effect
[40]	Gasteiger et al. (2021)	Computer, social robot, other technologies	7	3	L = positive effect SI = n/a
[47]	Khosravi and Ghapanchi (2016)	Computer, social robot, internet	7	4	L = n/a SI = positive effect
[54]	Poscia et al. (2018)	Telephone, smartphone, tablet, computer, social robot, videogame console, AR/VR system, messaging app, videoconference system, email, SNS, internet, other technologies	7	2	L = positive effect SI = n/a
[33]	Chen and Schulz (2016)	Telephone, smartphone, tablet, computer, videogame console, messaging app, videoconference system, email, SNS, internet, other technologies	6	2	L = unclear effect SI = positive effect
[34]	Choi and Lee (2021)	Smartphone, tablet, computer, social robot, videogame console, messaging app, videoconference system, email, SNS, internet, other technologies	6	2	L = unclear effect SI = positive effect
[35]	Choi et al. (2012)	Computer, email, internet, other technologies	6	2	L = positive effect SI = n/a
[39]	Gardiner et al. (2018)	Telephone, computer, social robot, videoconference system, SNS, internet, other technologies	6	4	L = positive effect SI = n/a
[44]	Ibarra et al. (2020)	Telephone, smartphone, tablet, computer, messaging app, videoconference system, email, SNS, internet, other technologies	6	4	L = positive effect SI = no effect
[38]	Franck et al. (2016)	Videogame console	5	2	L = positive effect SI = n/a
[55]	Shah et al. (2021)	Computer, messaging app, videoconference system, SNS, internet, other technologies	5	1	L = positive effect SI = n/a
[41]	Gorenko et al. (2021)	Telephone, computer, videoconference system, SNS, internet, other technologies	4	4	L = positive effect ^2^ SI = positive effect ^2^
[42]	Hagan et al. (2014)	Social robot, videogame console, videoconference system, internet, other technologies	4	4	L = unclear effect SI = n/a
[52]	Noone et al. (2020)	Smartphone, tablet, computer, videoconference system	3	1	L = unclear effect SI = unclear effect
[46]	Isabet et al. (2021)	Social robot	3	4	L = unclear effect SI = unclear effect
[29]	Brimelow and Wollin (2017)	Computer, social robot, videogame console, videoconference system, internet	2	4	L = unclear effect SI = n/a
				**Total**	Positive effect: L (12), SI (6) No effect: L (4), SI (3) Unclear effect: L (8), SI (5)
**Mixed-Methods Designs (10 Reviews)**					
**Ref.**	**Author(s)**	**CTs**	**Primary studies with Mixed-Methods Design**	**Quality Score**	**Effects on L/SI**
[40]	Gasteiger et al. (2021)	Computer, social robot, other technologies	10	3	L = positive effect SI = n/a
[43]	Heins et al. (2021)	Telephone, smartphone, tablet, computer, videogame console, messaging app, videoconference system, email, SNS, internet, other technologies	10	1	L = unclear effect SI = unclear effect
[32]	Chen et al. (2021)	Smartphone, videoconference system, email, SNS, internet, other technologies	8	4	L = positive effect SI = positive effect
[46]	Isabet et al. (2021)	Social robot	8	4	L = unclear effect SI = unclear effect
[28]	Baker et al. (2018)	Telephone, smartphone, tablet, computer, videogame console, videoconference system, email, SNS, internet, other technologies	3	4	L = n/a SI = unclear effect
[34]	Choi and Lee (2021)	Smartphone, tablet, computer, social robot, videogame console, messaging app, videoconference system, email, SNS, internet, other technologies	3	2	L = unclear effect SI = positive effect
[39]	Gardiner et al. (2018)	Telephone, computer, social robot, videoconference system, SNS, internet, other technologies	2	4	L = positive effect SI = n/a
[45]	Ibrahim et al. (2021)	Telephone, computer, email, internet, other technologies	2	4	L = no effect SI = unclear effect
[53]	O’Rourke et al. (2018)	Telephone, computer, social robot, videoconference system, email, internet, other technologies	1	4	L = n/a ^1^ SI = n/a ^1^
[54]	Poscia et al. (2018)	Telephone, smartphone, tablet, computer, social robot, videogame console, AR/VR system, messaging app, videoconference system, email, SNS, internet, other technologies	1	2	L = positive effect SI = n/a
				**Total**	Positive effect: L (4), SI (2) No effect: L (1), SI (0) Unclear effect: L (3), SI (4)

*Note*. Abbreviations: (Ref.) number according to reference list, (CTs) communication technologies, (L) loneliness, (SI) social isolation, (SNS) social networking site, (AR/VR) augmented reality/virtual reality system. ^1^ No general effects were reported. ^2^ The review included only interventions that had proven effective against loneliness and/or social isolation. n/a = the review did not include outcome measures for loneliness and/or social isolation. Reviews were classified according to the study design of the included primary studies. Reviews are presented in descending order within each category of study designs. The classification “positive effect” represents a decrease in loneliness and/or social isolation or an association with lower levels of loneliness and/or social isolation based on the conclusions of each review. Effects reported as “no effect” represent no significant variation in loneliness and social isolation based on the conclusions of each review. “Unclear effect” represents no general conclusion related to effects on loneliness and/or social isolation or conflicting results based on the conclusions of each review. Negative effects were not reported by any of the included reviews. *N* = 28 included research reviews.

**Table 5:** Effects of CT Use on Loneliness Among Older Adults as Reported by Included Reviews Grouped According to Study Design.

Study Design	No. of Outcome Measures for Loneliness (%)	Positive Effect (%)	No Effect (%)	Unclear Effect (%)
Qualitative	14 (22%)	9 (15%)	1 (2%)	4 (6%)
Quantitative-observational	16 (26%)	9 (15%)	1 (2%)	6 (10%)
Quantitative-experimental	24 (39%)	12 (19%)	4 (6%)	8 (13%)
Mixed methods	8 (13%)	4 (6%)	1 (2%)	3 (5%)
Total	62 (100%)	34 (55%) *	7 (12%) *	21 (34%) *

*Note*. Abbreviations: (No.) number. * Values do not add up to exactly 100% due to rounding. *N* = 28 included research reviews reporting 62 outcome measures of CT use regarding loneliness.

**Table 6:** Effects of CT Use on Social Isolation Among Older Adults as Reported by Included Reviews Grouped According to Study Design.

Study Design	No. of Outcome Measures for Social Isolation (%)	Positive Effect (%)	No Effect (%)	Unclear Effect (%)
Qualitative	10 (24%)	5 (12%)	1 (2%)	4 (10%)
Quantitative-observational	11 (27%)	5 (12%)	1 (2%)	5 (12%)
Quantitative-experimental	14 (34%)	6 (15%)	3 (7%)	5 (12%)
Mixed methods	6 (15%)	2 (5%)	0 (0%)	4 (10%)
Total	41 (100%)	18 (44%) *	5 (11%) *	18 (44%) *

*Note*. Abbreviations: (No.) number. * Values do not add up to exactly 100% due to rounding. *N* = 28 included research reviews reporting 41 outcome measures of CT use regarding social isolation.

## Data Availability

Not applicable.
